# *Strongyloides stercoralis* Hyperinfection in an HIV-Infected Patient Successfully Treated with Subcutaneous Ivermectin

**DOI:** 10.3390/tropicalmed3020046

**Published:** 2018-04-27

**Authors:** Paolo Antonio Grossi, Domenico Lombardi, Alessia Petrolo, Cristina Rovelli, Zaira Di Rosa, Giorgio Perriccioli, Agostino Rossi, Giulio Minoja, Francesco Scaglione, Daniela Dalla Gasperina

**Affiliations:** 1 Infectious and Tropical Diseases Unit, Department of Medicine and Surgery, University of Insubria, 21100 Varese, Italy; paolo.grossi@uninsubria.it (P.A.G.); domelomba@gmail.com (D.L.); alessiapet@libero.it (A.P.); cristina.rovelli02@universitadipavia.it (C.R.); zairadirosacb@gmail.com (Z.D.R.); d.dallagasperina@uninsubria.it (D.D.G.); 2 Clinical Pharmacology Unit, ASST Sette Laghi, 21100 Varese, Italy; giorgio.perriccioli@asst-settelaghi.it; 3 Laboratory of Medical Microbiology, ASST Sette Laghi, 21100 Varese, Italy; agostino.rossi@asst-settelaghi.it; 4 Intensive Care Unit, ASST Sette Laghi, 21100 Varese, Italy; giulio.minoja@asst-settelaghi.it; 5 Department of Oncology and Onco-Hematology, University of Milan, 20129 Milan, Italy; francesco.scaglione@unimi.it

**Keywords:** *Strongyloides stercoralis*, hyperinfection, HIV, parenteral ivermectin

## Abstract

A 39-year-old Ethiopian HIV-positive man with peripheral T-cell lymphoma developed *Strongyloides stercoralis* hyperinfection. The patient was initially treated with oral ivermectin for three weeks without response, most likely due to malabsorption because of concomitant paralytic ileus. Given the persistence of larvae in the body fluids, the worsening respiratory status and clinical malabsorption, veterinary parenteral formulation of ivermectin was administered. The very high plasma concentration of ivermectin achieved in the patient after parenteral administration led to a rapid improvement in his clinical condition and rapid disappearance of the parasite from biological samples, without any adverse reaction.

## 1. Case Report

A 39-year-old Ethiopian man, temporarily in Italy for professional reasons, with a recent diagnosis of human immunodeficiency virus (HIV) infection, on antiretroviral therapy (cART) with emtricitabine–tenofovir disoproxil fumarate and lopinavir/ritonavir, presented to the emergency department of our hospital with a month history of vomiting, abdominal pain, and diarrhea. His medical history included peripheral T-cell lymphoma located in the ethmoid and maxillary sinuses, treated with local radiation. 

On admission, he was febrile (up to 38 °C), with severe dehydration, tachycardia (heart rate of 100 beats/min) and hypotension (blood pressure of 90/50 mmHg). Initial laboratory tests showed an elevated white blood cell count of 14,790/mm^3^ with a normal eosinophil count of 260/mm^3^ (reference range <500/mm^3^), C-reactive protein level of 348.5 mg/L, hypoalbuminemia (1.9 g/dL), and acute renal failure (serum creatinine 5.45 mg/dL). Liver enzymes were slightly abnormal (aspartate aminotransferase 63 U/L and alanine aminotransferase 84 U/L) with a normal total bilirubin value (0.22 mg/dL). CD4 count was 402/mm^3^, and HIV-RNA was <20 copies/mL. A chest X-ray revealed bilateral areas of increased parenchymal density. The computer tomography (CT) scan of the chest and abdomen with intravenous contrast showed bilateral ground-glass opacities and areas of consolidation with pleural effusion, and small bowel wall thickening without distension. Blood, urine, and stool samples were collected for cultures. The patient was transferred to the intensive care unit (ICU), where supportive treatment and empiric broad-spectrum antibiotic therapy with trimethoprim/sulfamethoxazole, metronidazole, and ceftriaxone were started; cART was temporarily interrupted.

The following day, he developed altered mental status, progressive respiratory distress (PaO_2_ 61 mmHg), and persisting hypotension requiring vasopressors and endotracheal intubation. Due to severe stomach and abdominal distension, a nasogastric tube was placed, with a high gastric output (800–1200 mL/day). Bronchoscopy with bronchoalveolar lavage (BAL) for microscopic examination, cytology, and cultures was performed, with no significant finding on visual inspection. Microscopic examination of the BAL and stool showed parasitic organisms morphologically consistent with *Strongyloides* larvae. Serology for *Strongyloides stercoralis* by enzyme-linked immunoassay was positive. Administration of ivermectin 200 µg/kg daily by nasogastric tube was immediately started. Due to persistent fever, new urine and blood cultures were taken, and empiric antibiotic therapy was escalated to meropenem and vancomycin with discontinuation of ceftriaxone, metronidazole, and trimethoprim/sulfamethoxazole. 

**Figure 1:**
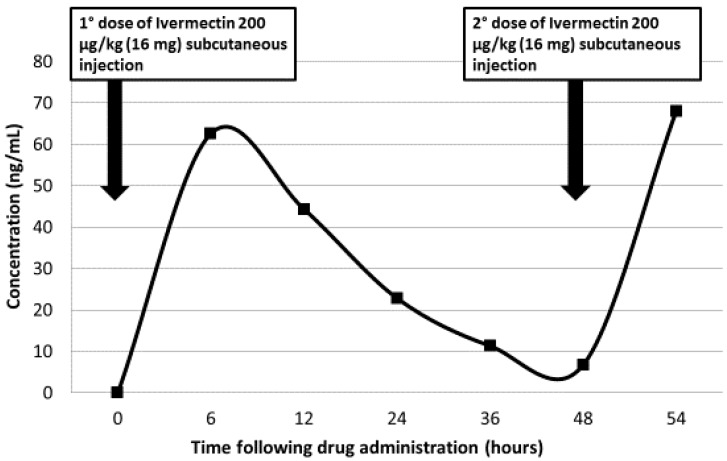
Plasma ivermectin concentrations after two parental administrations.

Despite intensive support care and antimicrobial therapy, the patient progressively deteriorated, developing paralytic ileus. After 22 days of antihelmintic treatment, BAL and stool specimens continued to demonstrate larvae and eggs of *S. stercoralis.* Given the persistence of larvae in the body fluids and malabsorption syndrome, emergency approval for the use of veterinary preparation of parenteral ivermectin (Ivomec^®^ 1% injection, licensed for veterinary use only) was obtained from the Local Therapeutic Committee, and subcutaneous injection of 16 mg (200 μg/kg) was administered. Subcutaneous administration was repeated at 48 h, without local or systemic reactions. After two days, larvae were no longer detected in the BAL and stool, and the patient’s symptoms improved. The concentrations of ivermectin in plasma were determined by high performance liquid chromatography (HPLC), using a previously-published extraction method [1]. The linear range of the calibration curve was 0.20–400 ng/mL from 0.20 mL plasma (Figure 1). 

Five days later, the patient was transferred from ICU to the infection diseases ward. All cultures (blood, urine, stool, BAL) were persistently negative for bacteria, mycobacteria, and fungi, and antibiotic therapy was therefore discontinued. A systemic lymphoma was excluded with hematological evaluation, maxillofacial CT scan, and positron emission tomography/computed tomography (PET/CT) scan. cART and rehabilitation program were started. At the 9-month follow-up visit, the patient was still asymptomatic with no medical problems, and direct stool examinations remained negative.

## 2. Discussion

*Strongyloides stercoralis* is one of the most important neglected soil-transmitted helminths, which is estimated to affect at least 370 million people worldwide [2]. *S. stercoralis* is endemic throughout the tropics and subtropics, and in limited areas in Europe and the United States [2]. It is a 2 mm long intestinal roundworm. Infection is acquired by percutaneous penetration of intact skin of filariform larvae that are present in infected soil. They are subsequently carried through the bloodstream to the lungs; then, they move up the respiratory tree, over the epiglottis and down to the small intestine, where female adult worms begin producing eggs, which later hatch in the mucosa, and larvae are then excreted with the feces. Most infected people are asymptomatic or have minor gastrointestinal or respiratory symptoms. Its medical importance primarily lies in its ability to produce overwhelming infection in immunocompromised subjects, as a consequence of its unique ability to replicate and increase in numbers without leaving its host [2,3].

Immunocompromised patients are at risk of developing hyperinfection syndrome and disseminated strongyloidiasis that can be fatal, mostly due to Gram-negative bacteremia and sepsis [3,4]. Severe strongyloidiasis typically follows corticosteroid therapy, but has also been described in patients with lymphoma, leukemia, human T-cell lymphotropic virus (HTLV) and HIV infection, malnutrition, chronic renal failure and end-stage renal disease, alcoholism, diabetes mellitus, advanced age, and in solid organ transplant recipients [2,3,4,5,6,7,8]. Therefore, it is mandatory to diagnose and treat chronic carriers, in order to prevent the hyperinfection syndrome if immunosuppressive treatment is planned [3,4,5,6,7,8]. With treatment, the mortality rate of hyperinfection is close to 60%; without effective treatment, it is likely to be around 100% [6]. Our patient had HIV infection diagnosed 3 months earlier (with a satisfactory CD4 count and undetectable HIV viral load) and a localized T cell lymphoma. Other potential risk factors for *S. stercoralis* infection, such as systemic lymphoma, other malignancies, or alcohol addiction, were investigated and excluded. Unfortunately, HTLV-1 and HTLV-2 serology was not performed during his hospital stay. However, considering that the *Strongyloides* hyperinfection, as well as the T-cell lymphoma are frequently associated with HTLV-1/2 infection, it would have been ideal to screen the patient for HIV/HTLV1/2-coinfection, especially since the immune status of the patient was rather good. According to the classical definition, the case described complies with a severe form of *Strongyloides* hyperinfection: exacerbation of gastrointestinal and pulmonary symptoms was seen, and increased numbers of larvae were found in stool and sputum. Larvae in non-disseminated hyperinfection are increased in numbers but confined to the organs normally involved in the pulmonary autoinfective cycle (i.e., gastrointestinal tract, peritoneum, lungs). The term ‘disseminated infection’ is often used to refer to migration of larvae to organs beyond the range of the pulmonary autoinfective cycle: larvae are also found in the central nervous system, kidneys, liver, and almost any other organ. In our patient, urine and other body fluids were not tested for *Strongyloides*, because neither brain nor abdominal CT showed any renal or encephalic injury. Moreover, the elevated value of serum creatinine at admission was immediately normalized after adequate hydration. The drug of choice for strongyloidiasis is ivermectin, which paralyses nematodes by increasing membrane permeability to chloride ions. Nevertheless, there are no subcutaneous formulations of these drugs approved for use in humans, and the appropriate dose, pharmacokinetics, and potential toxicity are unknown. However, in severe *Strongyloides* infection, there is often extensive larval infiltration of the small bowel that can cause a paralytic ileus and malabsorption, including impaired oral absorption of antihelmintic agents. This may explain why, despite the proper use of antihelmintic therapy with ivermectin via nasogastric tube, our patient did not show any clinical improvement. Conversely, the very high concentration of ivermectin (62.7 ng/mL) achieved after subcutaneous administration of ivermectin led to a rapid improvement in his clinical condition and rapid disappearance of the parasite from biological samples, without any adverse reaction. In our patient, we delayed the start of subcutaneous ivermectin because we thought that prolonged oral treatment would suffice. In addition, the use of veterinary drugs required an approval of the hospital Ethics Committee, and the supply was rather slow. Although data on the pharmacokinetics of subcutaneous ivermectin in humans are lacking, the use of veterinary subcutaneous ivermectin formulation in patients with hyperinfection syndrome and disseminated strongyloidiasis failing oral treatment should be considered promptly, as a life-saving option. In a recent report, Barret et al. summarized the published reports of subcutaneous ivermectin use (200 μg/kg daily or on alternate days) in 22 patients with severe strongyloidiasis or hyperinfection [9,10,11,12,13,14,15]. Treatment course length was generally dictated by clinical response, and varied from 3 to 11 doses. Only five case reports include serum ivermectin concentrations, although there is a lack of consistency in the timing of measurement of levels [11,12,13,14,15]. Serum ivermectin concentrations measured during treatment vary from 2 ng/mL after the first doses of 200 μg/kg [11], to 35.4 ng/mL after seven doses of 200 μg/kg [13]. There is no consensus on how subcutaneous ivermectin should be monitored, and the majority of pharmacokinetic studies are in farm animals. Ivermectin is protein-bound, so pharmacokinetics are altered by hypoalbuminaemia, a common result of severe *Strongyloides i*nfection. However, serum ivermectin concentrations and signs and symptoms of neurotoxicity should be carefully monitored [16]. Our patient had a complete resolution of symptoms, and direct examination of multiple stool samples remained negative. However, microscopic stool examination is inadequate, owing to low sensitivity to exclude an eventually persisting infection. A combination of concentration methods, like the Baermann method and/or agar plate culture (in particular, the technique described by Koga) with or without polymerase chain reaction (PCR), are more sensitive, but less commonly used techniques. PCR is not available at all diagnostic centers and, at the moment, is only applicable in research settings [4,17,18,19,20,21].

The reported case highlights the need for appropriate screening and eradication of *Strongyloides* to prevent potentially fatal episodes of hyperinfection in immunocompromised patients or in those with planned immunosuppressive therapy [22]. In addition, it confirmed the safety and efficacy of subcutaneous ivermectin. Further research is needed for addressing pharmacokinetic and ethical issues of veterinary drug use in humans.
